# Efficacy and safety of ACEI/ARB drugs in patients with COVID-19 combined with diabetes mellitus: A protocol for systematic review and meta-analysis of randomized controlled trials

**DOI:** 10.1097/MD.0000000000021723

**Published:** 2020-08-28

**Authors:** Yan Yang, Xiaoke Liu, Yan Liu, Yalin Chen, Yuan Tian, Xiaoxu Fu, Wen Zhong, Chunguang Xie

**Affiliations:** a Chengdu University of Traditional Chinese Medicine; b Affiliated Hospital of Chengdu University of Traditional Chinese Medicine, Sichuan, China.

**Keywords:** ACEI/ARB drugs, COVID-19, diabetes mellitus, protocol, RCTs, systematic review and meta-analysis

## Abstract

**Background:**

Novel coronavirus pneumonia (COVID-19) has become a worldwide epidemic, causing huge loss of life and property. Because of its unique pathological mechanism, diabetes affects the prognosis of patients with COVID-19 in many aspects. At present, there are many controversies about whether angiotensin-converting enzyme inhibitors/angiotensin receptor blockers (ACEI/ARB) should be used in the treatment of patients with diabetes mellitus and COVID-19 comorbidities. There is an urgent need to provide evidence for the use of ACEI/ARB through high-quality systematic evaluation and meta-analysis.

**Methods:**

We will search electronic databases including PubMed, Embase, the Cochrane Central Register of Controlled Trials (CENTRAL), Chinese Biomedical Literature Database, China National Knowledge Infrastructure, Chinese Science and Technology Periodical Database, and Wanfang database using keywords related to COVID-19, diabetes mellitus, ACEI/ARB drugs, and randomized controlled trials . We will manually search gray literature, such as conference proceedings and academic degree dissertations, and trial registries. Two independent reviewers will screen studies, extract data, and evaluate risk of bias. Data analysis will be conducted using the Review Manager software version 5.3.5 and stata 14.0 software for Mac. Statistical heterogeneity will be assessed using a standard chi-square test with a significance level of *P* < .10. Biases associated with study will be investigated using funnel plots.

**Results:**

This study will provide a high-quality synthesis of efficacy and safety of ACEI/ARB drugs in patients with COVID-19 combined with diabetes mellitus, providing evidence for clinical treatment of diabetes mellitus combined with COVID-19. And the results will be published at a peer-reviewed journal.

**Conclusion:**

Our study will draw conclusions on the efficacy and safety of ACEI / ARB drugs in patients with diabetes mellitus complicated with covid-19, so as to provide theoretical guidance for clinical practice of diabetes mellitus with covid-19.

**INPLASY registration number:**

INPLASY 202060111.

## 1 Introduction

In December 2019, the coronavirus disease of 2019 (COVID-19) caused by severe acute respiratory syndrome coronavirus 2 (SARS-CoV-2) was first discovered in Wuhan, China. SARS-CoV-2 belongs to the B lineage of the beta-coronaviruses and is closely related to the acute respiratory syndrome coronavirus (SARS-CoV) virus (80% homology).^[1–3]^ In March 11, 2020 the World Health Organization (WHO) announced it as a global pandemic.^[4]^ Many early studies have found that COVID-19 patients with chronic disease such as diabetes, hypertension, cardiovascular disease are more severe and have worse prognosis.^[5–9]^ Diabetes is a chronic inflammatory disease characterized by hyperglycemia and insulin resistance, combined with metabolic disorders, chronic inflammatory, coagulation state, and endothelial damage. Diabetes are closely related to the high morbidity of obesity, hypertension, and cardiovascular diseases. Diabetic cardiovascular complications and diabetic renal complications are important risk factors for severe complications in patients with COVID-19. Blood glucose and diabetes are independent risk factors for mortality and morbidity in SARS patients.^[10]^ Therefore, diabetes mellitus plays a central role in the influence of underlying diseases, metabolic disorders, immune abnormalities, and inflammatory status on COVID-19.

Angiotensin-converting enzyme 2 (ACE2) converts angiotensin II to angiotensin 1 to 7, so as to expand blood vessels and protect organs.^[11]^ So angiotensin-converting enzyme inhibitors (ACEI) or angiotensin receptor blockers (ARB) are commonly used by those with diabetes and hypertension.^[12]^ It has been reported that ACEI/ARB drugs can reduce the mortality and tracheal intubation rate of patients with viral pneumonia.^[13,14]^ ACEI/ARB drugs are considered to have significant immunomodulatory effects, which are beneficial for COVID-19 infection by reducing cytokines, reducing lung inflammation and systemic inflammatory response.^[15]^ While ACE2 is also one of the primary receptors for SARS-CoV invasion into the human body.^[16]^ Infection of circulating immune cells will increase the apoptosis of lymphocytes (CD3, CD4, and CD8) and lead to the decrease of lymphocytes. However, the degree of lymphocyte is related to the severity of SARS-CoV-2 infection.^[5–7,17]^ Diabetic patients with chronic inflammation are already faced with a higher risk of infection. Studies have found that the clearance rate of SARS-CoV-2 in diabetic patients is decreased. In addition, the use of ACEI/ARB drugs increases the level of ACE2, as well as the wide distribution of ACE2 in pancreas, lung, kidney, heart, and vascular endothelium, which may be an important cause of poor prognosis in patients with COVID-19 and diabetes mellitus. It has been worried that these drugs might affect negatively the outcome of COVID-19 patients.^[18]^

At present, there is no meta-analysis on the above arguments. It is necessary for us to make a real judgment on the effectiveness and safety of ACEI/ARB drugs for diabetes combined with COVID-19, so as to provide evidence for the use of ACEI/ARB drugs in treating diabetes mellitus combined with COVID-19.

## 2 Methods and analysis

### 2.1 Study registration

This study has been registered at INPLAYS (https://inplasy.com) with a registration DOI:10.37766/inplasy2020.6.0111. This systematic review protocol is reported in accordance with the Preferred Reporting Items for Systematic Review and Meta-analysis Protocols (PRISMA-P) checklist.^[19]^

### 2.2 Inclusion and exclusion criteria

#### 2.2.1 Study design

Randomized controlled trials (RCTs) can provide evidence about efficacy and safety of intervention, so they will be included in this systematic review.

#### 2.2.2 Participants

Patients confirmed with diabetes mellitus and COVID-19. Patients diagnosed with diabetes only or diagnosed with COVID-19 only were excluded without restriction of age, gender, course of disease. Without restriction of clinical stage, thus mild, moderate, or severe/critical case are appropriate. And there is no restriction of comorbidities.

#### 2.2.3 Intervention

ACEI/ARB drugs of any dose.

ACEI/ARB placebo or standard nursing.

#### 2.2.4 Outcomes

Primary endpoints.
Blood lymphocyte count
Time to clinical recovery.
All-cause mortality.
Secondary endpoints.
Incidence of severe complications
Blood glucose fluctuation

### 2.3 Study search

We will search electronic databases including PubMed, Embase, the Cochrane Central Register of Controlled Trials, Chinese Biomedical Literature Database, China National Knowledge Infrastructure, Chinese Science and Technology Periodical Database, and Wanfang database using keywords related to COVID-19, diabetes mellitus, ACEI/ARB drugs, and RCTs. They will be searched from their inception to October 1, 2020, with language limitation of English and Chinese. In addition, Google scholar and Baidu Scholar will be used to find out potential missing papers. To provide high-quality evidence, we only select articles with RCTs. The search strategy of PubMed is presented in Table 1.

**Table 1:**
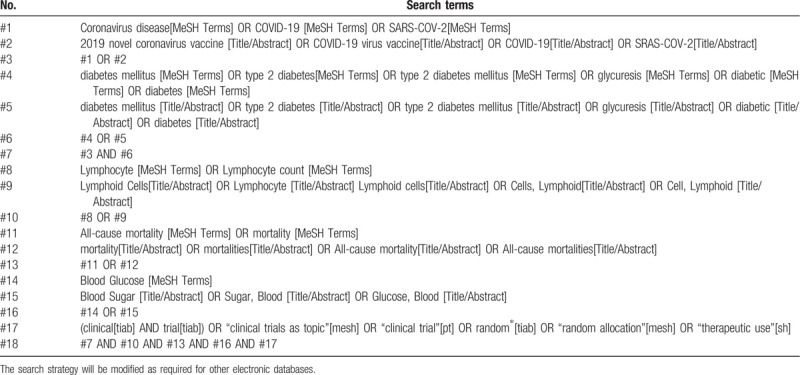
Search strategy for PubMed.

### 2.4 Study selection

**Figure 1:**
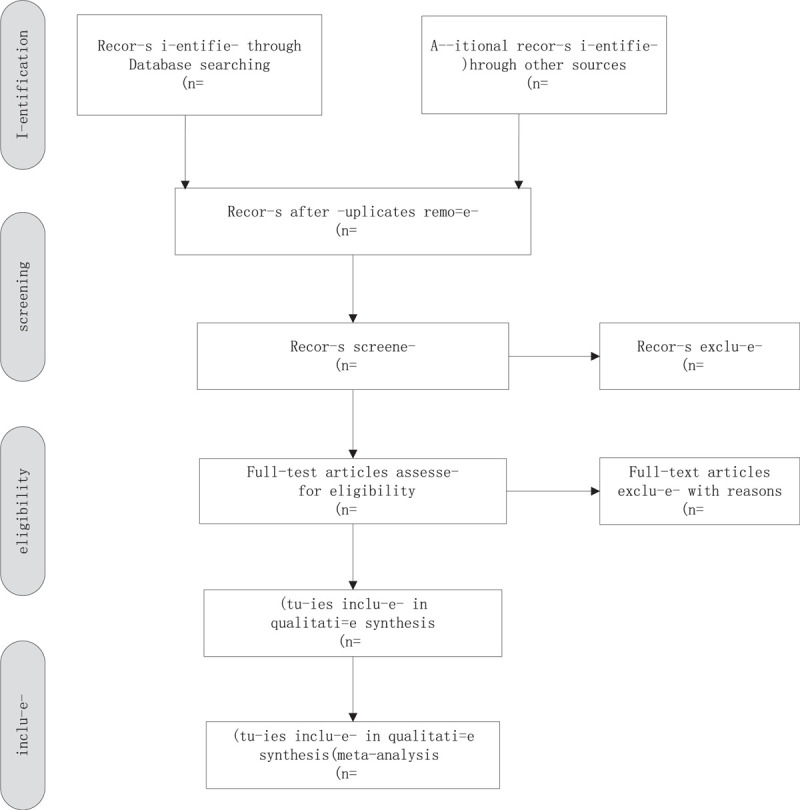
Flow chart of the study selection.

We will export the identified records in databases into EndNote X9 software and use this to identify duplicates. After removing duplicates, the retrieved records will be checked independently by 2 reviewers (YL and YC), who will apply the eligibility criteria based on the title and abstract. Where a study is potentially eligible, the full text will be obtained and checked independently by 2 reviewers (YL and YC) to identify the eligible studies. Any disagreements will be discussed and resolved in discussion with a third reviewer (YY) (Fig. 1).

### 2.5 Data extraction

Data extraction will be conducted by 2 independent authors (XL and HL) according to a prespecified form and checked by a third author (YY). The following data will be extracted: the first author's name, publication time, country, article title, article type, interventions in experimental and control group, course of treatment, severity of disease, number of patients in each group, ages and sex of patients, outcomes, and adverse effect.

### 2.6 Assessment of risk of bias

The Cochrane risk of bias tool^[20]^ will be used to assess the risk of bias for each included study. The risk of bias of each trial will be judged by 2 independent investigators as “‘Low,” “Some concerns,” or “High”based on the critical domains, including bias arising from the randomization process, bias due to deviations from intended interventions, bias due to missing outcome data, bias in measurement of the outcome, and bias in selection of the reported result. Disagreements will be resolved by discussion among the 2 investigators. If the disagreements persist, the third investigator will chip in as an arbitrator.

### 2.7 Data analysis

Data analysis will be conducted in Review Manager Version 5.3 and Stata 14.0 software. For the binary variable, the effect size will be represented as risk ratio and 95% confidence interval and a Der-Simonian-Laird method will be used to calculate them. Continuous data will be reported as an average difference from their 95% confidence intervals. I2 statistics will be used to test the statistical heterogeneity of the test. 75%, 50%, or 25% of I2 statistics indicate high, medium, and low heterogeneity, respectively. The statistical heterogeneity is considered substantial when *P* < .05 and I2 > 50% and the random-effect model will be applied to pool data. If there is no significant heterogeneity (*P* > .05 and I2 <50%), then the fixed-effect model will be used to calculate the effect size. If quantitative synthesis is not appropriate due to substantial heterogeneity, then systematic review will be conducted and the results will be presented with tables and figures. If there is a substantial heterogeneity and quantitative synthesis is not appropriate, the results will be presented in the form of tables and figures. Publication bias and small-study effects will be evaluated by funnel plot and statistically investigated by Egger test with *P* value boundary of.05.^[21]^

### 2.8 Investigation of heterogeneity

If there is substantial heterogeneity between studies, then we will conduct subgroup analysis to explore the heterogeneity.

### 2.9 Sensitivity analysis

To ensure the stability of the results, we will conduct sensitivity analysis of the results by excluding each of the studies included in the analysis one by one. If there is one or more very large study, we will repeat the analysis excluding them to determine how much they dominate the results.

### 2.10 Quality of evidence

Finally, this study will evaluate the quality of evidence for each outcome through grading of recommendations assessment, development, and evaluate system. RCTs will be defined as high-level evidence and observational studies as low-level evidence in grading of recommendations assessment, development, and evaluate system. Researchers can downgrade the quality of evidence in RCTs from a high level to a moderate or lower level depending on whether there are factors affecting the quality of evidence.

### 2.11 Patient and public involvement

There is no patient and public involved in this study.

### 2.12 Ethics and dissemination

Ethical approval is not needed for this meta-analysis. This study comprehensively evaluates the existing research evidence of ACEI/ARB drugs on the treatment of COVID-19 combined with diabetes and can provide evidence-based medical support for clinical workers. The results of our research will be published in a peer-reviewed journal.

## 3 Discussion

Like the evolution of other species, viruses are constantly changing and developing in nature. The outbreak and spread of SARS-CoV, SARS-CoV-2, or MERS-COV are great survival challenge for humans. In this case, ACEI/ARB drugs are particularly important due to their excellent inflammatory regulation, multisystem, and multiorgan protection contributions. Nevertheless, there is controversy because of lacking high-quality evidence. What we can do is to clarify the efficacy and safety of ACEI/ARB in the process of inflammatory infection complicated with multiple organ complications as soon as possible. To ensure the quality of the final evidence, we will only include RCTs studies. This may lead to insufficient number of included studies. In this regard, we will take appropriate measures to extend the search time. This is the first systematic review and meta-analysis about the above question. And we aim to provide reliable evidence for the treatment of diabetes combined with COVID-19 and provide guidance for clinical treatment.

### 3.1 Amendments

If any modification is required, we will update our protocol to include any changes in the entire research process.

## Author contributions

**Conceptualization:** Yan Yang, Yan Liu, Xiaoke Liu.

**Data curation:** Yan Yang, Yan Liu, Yalin Chen, Xiaoke Liu.

**Formal analysis:** Yan Yang, Wen Zhong.

**Funding acquisition:** Yuan Tian.

**Methodology:** Yan Yang, Xiaoke Liu.

**Resources:** Xiaoke Liu, Xiaoxu Fu, Chunguang Xie.

**Software:** Yan Yang, Yan Liu, Yalin Chen.

**Supervision:** Xiaoke Liu.

**Writing – original draft:** Yan Yang.

**Writing – review & editing:** Yan Yang, Xiaoke Liu, Chunguang Xie.
